# Post Vaccinal Temporary Sensorineural Hearing Loss

**DOI:** 10.3390/ijerph15081780

**Published:** 2018-08-19

**Authors:** Federica De Marco, Donato Pompeo De Cesare, Francesco Di Folco, Francesco Massoni, Gianfranco Tomei, Natale Mario Di Luca, Carmina Sacco, Francesco Tomei, Ricci Serafino

**Affiliations:** 1 Department of Anatomy, Histology, Medical-Legal and the Orthopedics, Specialty School of Occupational Medicine, Unit of Occupational Medicine, Sapienza University of Rome, 00161 Rome, Italy; federica.demarco@uniroma1.it (F.D.M.); dondece@icloud.com (D.P.D.C.); serafino.ricci@uniroma1.it (R.S.); 2 Policlinico Militare Celio, 00184 Rome, Italy; difolco.francesco@libero.it; 3 Department of Anatomy, Histology, Legal Medicine and Orthopaedics, Sapienza University of Rome, 00161 Rome, Italy; francesco.massoni@uniroma1.it (F.M.); natalemario.diluca@uniroma1.it (N.M.D.L.); carmina.sacco@uniroma1.it (C.S.); 4 Department of Psychiatric and Psychological Science, University of Rome “Sapienza”, Piazzale Aldo Moro 5, 00185 Rome, Italy; gianfranco.tomei@uniroma1.it; 5 Spin off Sipro, Via Stimigliano 5, 00199 Rome, Italy

**Keywords:** vaccine, adverse reaction, hearing loss

## Abstract

In our systematic research we identified four studies concerning the onset of neurological adverse events following vaccination and two excluding this association. A 33-year-old Italian man, belonging to the Italian Army was hospitalized because he suffered from vertigo, nausea and sudden right hearing loss not classified (NDD), that set in 24 h after the administration of tetanus-diphtheria and meningococcal vaccines. Some neurological events arising after vaccination are very difficult to treat. In our case, the functional recovery on low and medium frequencies was possible about 6 months after the morbid event.

## 1. Introduction

In the case reported here, the patient developed hearing loss, dizziness and nausea within 24 h after receiving the meningococcal and tetanus-diphtheria vaccine.

## 2. Case Report

A 33-year-old Italian man of Indo-European descent, in military service since 2001, was admitted to the Policlinico of an Italian city because of sudden hearing loss NDD, within 24 h of receiving two vaccines: intramuscular tetanus and diphtheria vaccines (Ditanrix 0.5 mL) and subcutaneous meningococcal polysaccharide vaccine (Mencevax 0.5 mL). After informed consent, the subject had underwent two vaccinations: on the right arm meningococcal vaccine and on the left arm tetanus-diphtheria vaccines. Nothing relevant was observed in his family and remote pathological history. He also completed a questionnaire to exclude conditions that could be the reasons for temporary or permanent contraindication to the aforementioned vaccinations. The following conditions were excluded:

1. Concomitant intake of other drugs, homeopathic, nutritional supplements, products based on medicinal plants.
2. A history of adverse reactions to previous vaccination.
3. Suspected or documented allergies to eggs, feathers, chicken meat, duck meat, beef/gelatine, formaldehyde, antibiotics (neomycin, streptomycin, kanamycin, polymyxin B, mercurial compounds).
4. Recent positive history of fever conditions > 38°, airway disorders, diarrhea, intake of any treatments in the last 72 h (antibiotics, NSAIDs, cortisones, aspirin, antimalarial drugs), blood transfusion in the last 6 months, and administration of immunoglobulins.

Twenty four hours after the vaccinations, the subject complained of dizziness, nausea and right hearing loss due to NDD, and in emergency department he underwent audiometric examination, tympanogram with a diagnosis of “severe right perceptual deficit”. Following the persistence of the symptoms despite the pharmacological medical was admitted to the otorhinolaryngology department. During the few days of hospitalization, many investigations were performed such as brain NMR to exclude a vascular origin of the hearing loss or other causes, as well as other audiometric and otorhinolaryngology examinations. Since the second day of admission until discharge, the patient underwent the following therapy: Bentelan (betamethasone 21-sodium phosphate) 4 mg 1 fl e.v. twice daily, vitamin B12 1 fl i.m. once a day, omeprazole and Trental (pentoxifylline) 1 fl i.m. 1 once a day, carbogen for 30 min every two hours. After 11 days he was discharged with a diagnosis of “improvement of sudden hearing loss” and the following home treatment: Bentelan 1 cpr/day for 30 days, Deflan (deflazacort) 25 mg 1cpr/day × 2 for 3 days, then ½ cpr × 2 for 3 days and ½ cpr × 1 for 4 days, Lucen (esomeprazole) 20 mg 1 cpr for 10 days.

We report the results of the audiometric tests at the time +1 (24 h after vaccination), +2 (during hospitalization, 48 h after vaccination), at discharge (+9) and after about 6 months (+159) (Table 1).

The last audiometry performed approximately 6 months after the morbid event shows a functional recovery on low and medium frequencies. The subject enjoys good health at present. He expressed his consent to the therapy and to the elaboration of this study.

The subject chose to make personal information available after learning that its personal data would be classified as critical data. They also accepted the processing of anonymous and collective data, analyzed using scientific methods and for scientific purposes based on the declarations of the Helsinki Declaration.

## 3. Discussion

Sudden neurosensory hearing loss (SHL) is usually idiopathic but in some cases it may be associated with infections, vasculitis, tumors, some genetic diseases and cardiovascular diseases [1,2]. Reported cases of hypoacusis resulting from the vaccination have suggested a possible correlation [3,4,5]. The cause of our patient's neurosensory hearing loss remains unknown despite the many specialist investigations in different hospital departments such as RMN of the brain as well as other audiometric tests and otorhinolaryngology examinations to exclude a vascular origin or other causes. The close association between the vaccinations and the onset of hearing loss suggests, however, that it may be the result of an adverse reaction to vaccines. A variety of adverse reactions to vaccines is certainly known: anesthesia, paraesthesia, chronic arthritis, encephalopathy, hypotonia/hypersensitivity, manifestations immediate hypersensitivity, meningitis, brachial neuritis, acute flaccid paralysis, persistent cry, thrombocytopenic purpura, anaphylactic shock, Guillan-Barrè/polyradicoloneurite, encephalitis and convulsions [6,7,8,9,10,11]. However, the data on possible neurological collateral effects following vaccination in the literature are few and inconsistent. Some studies showed that the MPR vaccine could cause sensorineural hearing loss, following the development of an acoustic neuritis with fibrosis and direct cytolytic damage with atrophy of the organ of Corti [4,12,13,14]. A correlation between the administration of vaccines and neurological alterations is possible, according to a study conducted in 2012, Schulze [15] describes the case of a 44-year-old military patient, who developed epileptic seizures after taking multiple doses of vaccine against rabies, abdominal typhus, yellow fever and meningococcus. He received his third dose against Japanese encephalitis (JEV) four weeks after the previous vaccination and 24 h later he developed the first epileptic fit after which he was admitted to the Neurology Department of the Guestrow Hospital. His conditions has not yet stabilized despite the various pharmacological treatments,. The occurrence of convulsions can be mediated by the immune system [16]. JEV is a vaccine that can cause a wide variety of complications, including encephalitis [17,18,19] although no neurological secondary effects to its administration have been reported to date. Moreover, as it is in the case of our patient, it is not clear whether the episodes are related to the isolated administration of a single vaccine or to the combination with the others. A common and indisputable scenario, however, is the close temporal relationship between the neurological manifestations and the administration of polysaccharide vaccines or derived from neurotropic viruses/bacteria. A post-vaccinal correlation seems plausible (in our case the vaccination against meningococcus, tetanus and diphtheria) because despite extensive investigations no alternative etiology has been identified. Like in the study conducted in 2012 by Cheng [20], also in our case following the vaccine administration there was a damage to a cranial nerve. The exact pathophysiology is not known, although the hypotheses of possible autoimmune mechanisms and direct viral invasion could explain the pathophysiology underlying the immunization of the affected nerves. These hypotheses include the damages from autoimmune mechanisms or demyelination linked to viral invasion with consequent localized arteritis or a particular genetic predisposition that could increase susceptibility to such paralysis [21,22,23]. The hypothesis that this could be caused by additives in the vaccine is unlikely. In fact, the neomycin which is used as a preservative to prevent bacterial contamination both in MPR and in varicella vaccines, has only shown a sort of contribution to the genesis of local or systemic allergic anaphylactic reactions [24].

The clinical course of post-vaccine SHL can be variable, from unilateral forms to bilateral forms with an involvement that can be partial, severe and often permanent [3,4,13,25,26]. However, it is not always possible to verify to association; in a study in 2016 Baxter et al. [27] analyzed all cases from 2007 to 2013 of first SHL diagnosis in a specific time interval from the administration of vaccines against tetanus, pertussis, diphtheria, varicella and inactivated influenza trivalent and did not find any significant evidence correlating the two events. This could highlight a possible genetic role of particular predisposition and greater sensitivity of certain subjects with causing the development of a toxic-autoimmune damage or demyelination of the nerve bundles and subsequent neurosensory hearing loss. In the literature, however, it to date there is not sufficient scientific evidence indicating sudden hypoacusis as an adverse event to vaccination. In our case there is also no evidence that the subject was hypersensitive/allergic to any compound (it is not in the anamnesis) nor that the vaccine was administered incorrectly; as a member of the army and he had already tolerated multiple vaccination in short intervals of time for the same infectious agents. Considering this last detail we thought over the most probable etiology for the hearing impaired. A possible explanation could be linked to the genesis of a mechanism of central nervous system sensitization, probably established during the previous vaccinations, and to the consequent toxic-autoimmune damage of nerve cells (for subsequent recalls). In our case, in 6 months time the functional hearing recovery was probably restored after the resolution of the neuronal insult with a return to an adequate electrical-receptorial activity.

## 4. Conclusions

Vaccinations are an effective means of preventing infectious diseases. Despite the complete control measures, the precise instructions for the application of vaccines and the clinical studies, adverse reactions cannot be completely excluded [28,29]. In the case presented here, the sudden onset of transient sensorineural hearing loss is more likely to have been triggered by a post-vaccinal damage rather than onset of a new pathology (however excluded through extensive instrumental investigations). Despite the many specialist investigations in various hospital department and after excluding possible related pathologies, the cause of our patient's neurosensory hearing loss remains unknown. The short period of time between the vaccinations and the onset of the hearing loss suggests, however, that it may be the result of an adverse reaction to vaccines. Fortunately the functional recovery was achieved after about 6 months and he subject is at present in good health. People serving in the army and employed abroad often receive multiple vaccinations in short time intervals before reaching in the operating theaters although all vaccinations are given in accordance with the regulations in force, with the national authorities and the international directives and in the routine clinical practice they are generally associated with a low incidence of complications, however some risks, as in our case, cannot be completely excluded.

## Figures and Tables

**Table 1:** The audiometric tests at the time +1 (24 h after vaccination), +2 (during hospitalization), at discharge (+9) and after about 6 months (+159).

	Time (+1)	Time (+2)	Release (+9)	6 Months (+159)
125 Hz		40 db	10 db	10 db
250 Hz	60 db	50 db	10 db	10 db
500 Hz	70 db	90 db	10 db	10 db
1000 Hz	80 db	120 db	25 db	10 db
2000 Hz	80 db	120 db	55 db	20 db
3000 Hz		120 db	65 db	60 db
4000 Hz	115 db	65 db	60 db	
6000 Hz		80 db	70 db	
8000 Hz		90 db	70 db	
